# Clustering single-cell multi-omics data via weighted distance penalty and adaptive consistent graph regularization

**DOI:** 10.1371/journal.pcbi.1014110

**Published:** 2026-04-03

**Authors:** Wei Zhang, Yue Yu, Xiaoying Zheng, Juan Shen, Yuanyuan Li

**Affiliations:** 1 School of Mathematics and Physics, Wuhan Institute of Technology, Wuhan, Hubei, China; 2 School of Science, East China Jiaotong University, Nanchang, Jiangxi, China; University College London, UNITED KINGDOM OF GREAT BRITAIN AND NORTHERN IRELAND

## Abstract

Recent advancements in single-cell multi-omics technologies have significantly improved our ability to explore cellular heterogeneity at an unprecedented resolution. These innovations enable the simultaneous profiling of genomic, transcriptomic, proteomic, and epigenetic data at the single-cell level, providing comprehensive insights into cellular states and their regulatory mechanisms. However, integrating multi-omics data remains challenging due to its high dimensionality, technical noise, and biological complexity. To address these challenges, we introduce scWDAC (single-cell weighted distance adaptive clustering), a novel clustering method for single-cell multi-omics data. scWDAC utilizes a weighted distance penalty and adaptive graph regularization to effectively integrate multiple omics layers. Key innovations of scWDAC include using a weighted distance penalty to capture cell-to-cell similarities across different omics modalities, and applying adaptive graph regularization on a consensus matrix to enforce cross-modal consistency. The framework optimizes both global consistency and local accuracy, ensuring a robust exploration of cellular structures across all omics layers. The effectiveness of scWDAC is evaluated through extensive experiments on ten paired single-cell multi-omics datasets. The results demonstrate that scWDAC outperforms existing clustering methods in terms of clustering accuracy, robustness to noise, and biological interpretability.

## Author summary

Recent advancements in single-cell high-throughput technology have transformed single-cell multi-omics, enabling researchers to study cellular heterogeneity with high resolution. This progress has significantly improved our understanding of complex biological systems and diseases. Single-cell multi-omics data, including genomics, transcriptomics, proteomics, and epigenetics, provides a comprehensive view of cellular states and gene regulatory mechanisms. However, its analysis remains challenging due to high dimensionality, noise, and complexity. To address these challenges, we present scWDAC, a novel clustering algorithm for single-cell multi-omics data. scWDAC integrates omics layers by employing weighted distance penalties and adaptive graph regularization. It captures cell-to-cell similarities across layers, minimizes low-rank approximations, and enforces cross-modal consistency through consensus matrix regularization. By optimizing both global consistency and local accuracy, scWDAC uncovers robust cellular structures.

## Introduction

Single-cell sequencing technology has revolutionized the study of biological processes and disease mechanisms at individual cell resolution [1]. This advancement has profoundly enhanced our understanding of complex biological systems and diseases, including cancer, immune disorders, and chronic conditions [2,3]. In particular, techniques such as scRNA-seq [4], scATAC-seq [5], scDNA-seq [6], and sci-CAR-seq [7] now enable the profiling of multiple molecular layers within the same cell, opening new avenues for deciphering cellular heterogeneity. A central task in single-cell data analysis is clustering—grouping cells based on their multidimensional characteristics to reveal underlying cellular subtypes [8]. Clustering is crucial for revealing heterogeneity within cell populations and lays the foundation for subsequent analyses, including the identification of novel cell types, inference of cellular trajectories, and mapping of complex cellular landscapes [9,10].

Traditionally, clustering methods for single-cell data [11–19] have primarily focused on scRNA-seq data, leading to significant progress in identifying cellular heterogeneity. For instance, Wang et al. [12] introduced SIMLR, a multi-kernel learning framework for clustering scRNA-seq data. Similarly, Zhang et al. [14] proposed SCCLRR, a method that captures both global and local features of scRNA-seq data to accurately detect cell types by learning a robust similarity matrix. Wu et al. [15] developed DRjCC, which combines dimensionality reduction with non-negative matrix factorization for cell type identification. However, these methods are limited by their reliance on scRNA-seq, which captures only the transcriptional layer of cellular activity. Consequently, they are highly sensitive to technical artifacts in scRNA-seq data—such as dropout events, amplification biases, and temporal expression fluctuations. This may lead to misclassification of functionally similar or transitional cell states that possess distinct epigenetic or genomic profiles.

The emergence of single-cell multi-omics technologies now enables the simultaneous analysis of multiple molecular layers–such as genomics, transcriptomics, proteomics, and epigenomics–at single-cell resolution. Notably, sci-CAR-seq complements scRNA-seq by incorporating chromatin accessibility data, thereby offering a more comprehensive understanding of gene regulation at both the transcriptomic and epigenomic levels. Integrating these diverse data types enables researchers to gain a more comprehensive understanding of cellular behavior, which may lead to the discovery of novel biomarkers, biological mechanisms, and therapeutic targets [20,21]. However, although these multi-omics datasets offer deeper biological insights, they also pose significant challenges related to data integration, noise reduction, and computational scalability. For example, data from scRNA-seq, scATAC-seq, and scDNA-seq provide complementary but incomplete insights into cellular function. Therefore, there is a pressing need for advanced methods to effectively integrate and analyze single-cell multi-omics data. These methods should preserve the inherent relationships between different omics layers while accounting for biological variability in computational systems biology.

In recent years, numerous computational methods have been developed to address the challenges of analyzing single-cell multi-omics data [22–31,32,33]. These methods can be broadly categorized into paired (multi-omics data from the same cell) or unpaired data (multi-omics data from different cells). Methods designed for unpaired data aim to project multiple modalities into a common latent space or leverage transfer learning to fill in missing modalities. For example, Seurat V3 [34] integrates scATAC-seq with scRNA-seq by transforming datasets into a shared space via “anchors.” UnionCom [22] uses a topology-preserving algorithm to align multi-omics data, preserving both global and local relationships between cells. However, this approach may struggle to scale with large datasets. uniPort [35] embeds different omics datasets into a shared latent space and integrates multi-omics via coupled variational autoencoders and unbalanced optimal transport.

For paired data, where different modalities are profiled from the same set of cells, methods such as MOFA+ [25] combine single-cell RNA-seq, ATAC-seq, and DNA methylation to identify latent factors based on non-negative matrix factorization (NMF), effectively capturing cellular heterogeneity. Its primary strength lies in handling diverse omics data in an unsupervised manner, although it only considers the linear relationships between the omics layers and requires careful hyperparameter tuning to avoid overfitting. Additionally, scHoML [31] applies a multimodal high-order neighborhood Laplacian matrix optimization framework to enhance clustering performance and provide insights into cellular states. GRMEC-SC [33] incorporates graph-based regularization to preserve the data’s intrinsic structure, ensuring more accurate and robust clustering results. Both JSNMF [28] and CCNMF [36] are based on NMF. JSNMF assumes that latent variables for different omic types are distinct and integrates the corresponding latent factorized matrices through consensus graph fusion. It is specifically designed for dual-omics data. In contrast, CCNMF models the shared underlying clonal structure and the general concordance between cellular expression levels and copy number states by maximizing global concordance across different omic layers. sLMIC [37] utilizes low-rank and exclusivity constraints to decompose the self-representation of cells into shared and specific features, offering an effective approach to integrating different omic layers. Although graph-based methods have shown promising performance in single-cell multi-omics integration, most approaches rely on intuitive assumptions about shared features or consensus terms across omics layers. Crucially, they often overlook structural consistency and the complex relationships between features from different omics modalities.

**Fig 1:**
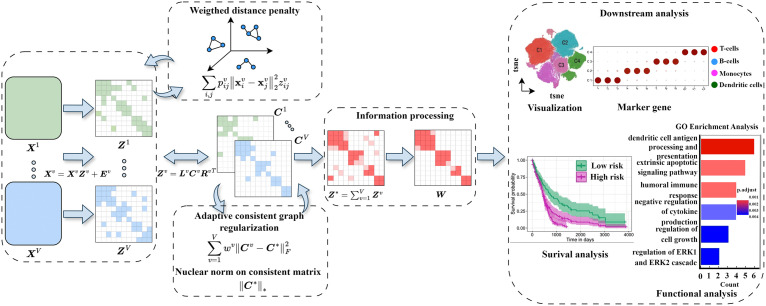
Framework of scWDAC. First, scWDAC employs a weighted distance penalty strategy combined with LRR to capture both local and global structures across multiple omics, thereby enhancing the representation of data. It then aligns the representation matrices via a three-factor decomposition, preserving the critical information through the core matrices $C^{v}$ for each omic. Additionally, adaptive consistent graph regularization is applied to enforce cross-modal consistency. Next, an adaptive information simplification strategy is applied to $Z^{*}$ to reduce redundant information and noise. Finally, downstream analyses, including *t*-SNE visualization, gene marker identification, functional analysis, and survival analysis, are performed based on the predicted results.

To address these limitations, we propose scWDAC, an innovative computational framework that effectively captures both global structures and nonlinear local relationships in single-cell multi-omics data. scWDAC employs adaptive graph regularization to enhance clustering accuracy while ensuring cross-omics consistency, thereby preserving the intrinsic biological features across different molecular layers. A schematic overview of the scWDAC framework is presented in Fig 1.

In summary, the primary innovations and contributions of scWDAC are:

- scWDAC integrates Gaussian kernel-based weighted distance penalties to capture local nonlinear relationships, combined with low-rank representation (LRR) to preserve global structural patterns. This approach effectively addresses both fine-scale cellular variations and broader system-level consistency in multi-omics data.
- The method introduces an innovative three-factor decomposition with adaptive graph regularization that maintains omics-specific information while enforcing biologically meaningful consistency across different molecular layers, overcoming key limitations in current multi-omics integration approaches.
- Extensive validation across ten benchmark datasets demonstrates that scWDAC consistently outperforms current methods in clustering accuracy and robustness.

## Results

### Comparison of clustering results

**Fig 2:**
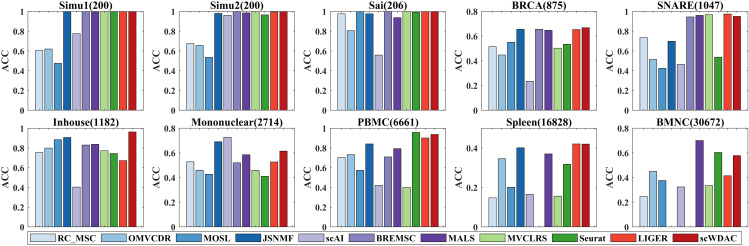
Comparison of clustering performance across ten datasets. Bars represent the average ACC over 10 independent runs. Missing bars indicate methods that exceeded the 60-hour runtime limit or available memory.

**Fig 3:**
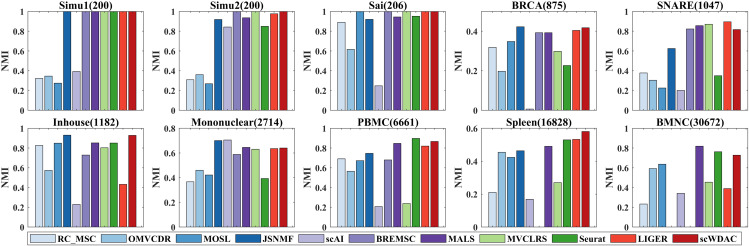
Comparison of clustering performance across ten datasets. Bars represent the average NMI over 10 independent runs. Missing bars indicate methods that exceeded the 60-hour runtime limit or available memory.

In this section, we evaluate the clustering performance of scWDAC by comparing it with advanced clustering methods using three metrics: Accuracy (ACC) [38], Normalized Mutual Information (NMI) [39], and K-Nearest Neighbors Accuracy (KNA). Detailed descriptions of these metrics are provided in Section 2 of the S1 File. As shown in Figs 2 and 3, scWDAC attains perfect scores in both ACC and NMI for the Simu1, Simu2, and Sai datasets. For the BRCA, Inhouse, and Spleen datasets, scWDAC outperforms all other compared methods. On the remaining four datasets, scWDAC demonstrates strong competitiveness; for example, on the PBMC dataset, it ranks second only to Seurat. Althrough the JSNMF performs well in terms of ACC and NMI metrics under servel datasets, this method is designed for dual-omics data and in cases when the number of omics excedds two, we select the best dual-omics result as the final output. Comprehensive numerical results of ACC and NMI are provided in S1 and S2 Tables.

Analysis of the clustering results revealed the complementary nature of ACC and NMI. ACC is sensitive to correct assignment of samples in large clusters, as misclassifications in populous groups heavily penalize overall accuracy. In contrast, NMI, which is based on information theory, provides a more balanced assessment by considering mutual information between cluster distributions, thereby remaining relatively equitable toward clusters of all sizes.

**Fig 4:**
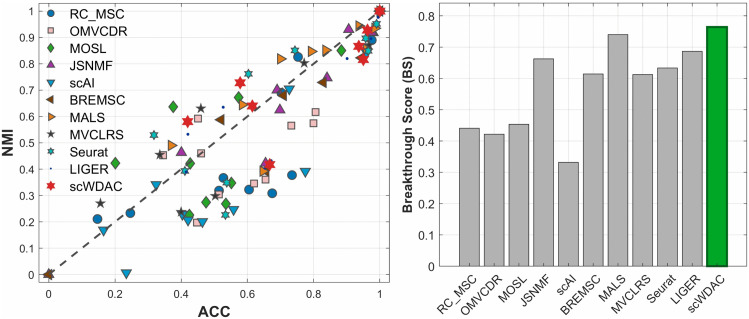
Evaluation of clustering performance trade-offs. (left) Scatter plot showing the relationship between ACC and NMI for each method across datasets. (right) Bar plot comparing BS values for each method, which quantifies the ability to balance both metrics.

To quantitatively assess a method’s ability to simultaneously achieve high clustering accuracy and balanced cluster distributions across diverse datasets, we introduced the Breakthrough Score (BS) (details provided in Section 2 of the S1 File). The scatter plot of ACC versus NMI and the bar plot of BS values for each method are shown in Fig 4. scWDAC achieved the highest BS score (0.764), outperforming all baseline methods. This result confirms scWDAC’s unique capability to overcome the ACC-NMI trade-off.

Due to the fact that ACC and NMI primarily focus on evaluating global clustering agreements, they do not fully capture the preservation of local structures. Here, we introduce a new metric KNA designed to characterize the effectiveness of local structure preservation. S3-S5 Tables show the KNA metric results for scWDAC at various values of *k*. scWDAC consistently achieves the best performance on the Simu1, Sai, BRCA, and Inhouse datasets. On the Simu2 and Mononuclear datasets, scWDAC ranks just below JSNMF and scAI, respectively. scWDAC performs slightly inferior to MALS and MOSL on SNARE and PBMC datasets, it achieves the best average KNA score across all datasets compared to the other methods. Notably, JSNMF also exhibits a strong ability to preserve local structural information. This advantage arises from the use of a graph Laplacian regularization term, which enables the model to capture and retain the intrinsic local geometric structure of high-dimensional data. All experiments are conducted with the number of clusters set to match the true number of classes in the datasets. The results are obtained on a Windows 10 system with an Intel Xeon E5-2686 V4 (2.3GHz) [Dual CPU] and 256GB RAM, using MATLAB 2022b and R 4.5.1.

Overall, our proposed method achieves the best overall clustering performance across all datasets. This is evidenced by its highest average scores in ACC and NMI metrics (S1 and S2 Tables) and superior average scores on local KNA metric (S3–S5 Tables), which collectively demonstrate its stability and robustness. These results suggest that integrating both linear and nonlinear information significantly enhances clustering performance. Additionally, the adaptive consistency graph regularization strategy, which enforces cross-modal consistency, effectively improves the model’s robustness across diverse datasets.

We present a visualization of the clustering results in Section 4 of the S1 File and S1 Fig on page 5 to provide an intuitive evaluation of the new method’s performance. Further assessment is conducted by comparing the heatmap of the similarity matrix generated by our method with those produced by other methods. S2 Fig depicts the block diagonal structure of the similarity matrices for both our method and the compared methods, based on the Sai and SNARE datasets. Additionally, we analyze the sensitivity of the parameters in Section 5 of the S1 File. The results in S3 and S4 Figs demonstrate that scWDAC is relatively stable and insensitive to parameter variations across most test datasets.

### Marker gene identification and functional enrichment analysis

Marker genes play a crucial role in understanding cellular heterogeneity and transcriptional regulation. Identifying marker genes is a key step in cell type annotation. In this section, we used the cosine similarity-based marker gene identification method (COSG) [40] to identify significant marker genes. This method evaluates gene importance by integrating the gene expression matrix with predicted cell labels, ranking the genes in descending order of significance. The top-ranked genes are considered important marker genes, as their potential roles in cellular processes are often reflected in their high expression levels and unique expression patterns.

**Fig 5:**
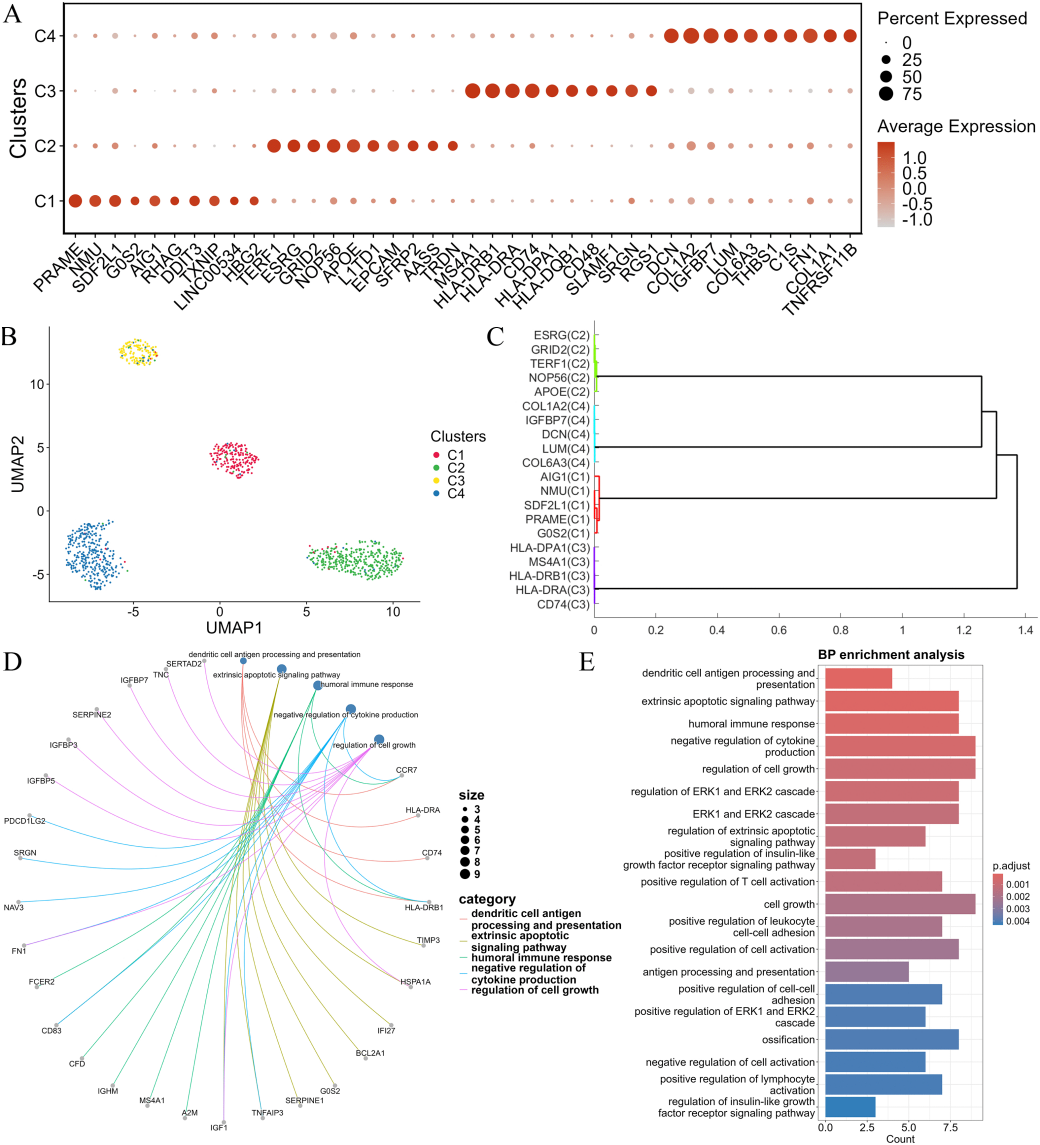
Downstream analyses of the SNARE dataset. (A) Bubble plot of marker genes across cell types. (B) UMAP plot ofvisualizing the distinct clusters of cells. (C) Pairwise correlation of averaged gene expression values for each cluster. (D) Correlations between marker genes and biological processes. (E) Top 20 enriched BP categories, ranked by *p***-value**.

Fig 5A presents a bubble plot of the top 10 marker genes identified in each cell cluster from the SNARE dataset. For example, PRAME has been identified as a key inhibitor of the retinoic acid receptor (RAR) signaling pathway. In leukemia cell line models, PRAME expression interferes with the normal regulation of cell proliferation and differentiation by retinoic acid. Specifically, PRAME inhibits cell differentiation and promotes cell proliferation by blocking RAR signal transduction [41]. Therefore, antibodies targeting PRAME may serve as potential therapeutic targets for leukemia. Long non-coding RNAs (lncRNAs) are essential for the self-renewal and maintenance of pluripotency in human embryonic stem cells (hESCs). The lncRNA ESRG is highly expressed in undifferentiated hESCs, where it binds and stabilizes the HNRNPA1 protein, regulating the alternative splicing of TCF3 and influencying CDH1 expression, thus maintaining hESC self-renewal and pluripotency [42]. The marker genes identified by the scWDAC method exhibit differential expression across their respective cell clusters, with most matching entries in the CellMarker database [43]. Although some identified marker genes are not recorded in CellMarker, the experimental results indicate their elevated expression levels in different clusters, suggests that they may represent novel merker candidates. To further assess the performance of scWDAC in marker gene identification, we performed comparative experiments in Section 6 of the S1 File.

Fig 5B shows a UMAP visualization of the SNARE dataset containing K562 cells (C1), H1 cells (C2), GM12878 cells (C3), and BJ cells (C4). To further validate the reliability and relationships among the identified marker genes, we select the top 5 marker genes from each cluster in the SNARE dataset. We then analyze the pairwise similarities between the average gene expression values within each cluster and construct a dendrogram based on these similarity scores using hierarchical clustering to identify new clusters. The results shown in Fig 5C demonstrate that marker genes from the same cell type are perfectly reclassified in the SNARE dataset. These results indicate that the marker gene predictions identified by combining scWDAC and COSG provide valuable insights for downstream analysis and investigations into cellular regulatory mechanisms.

To investigate the inherent relationships between genes and biological processes, we analyze the enrichment of marker genes within five typical biological processes (BP). As shown in Fig 5D, this analysis reveals a close association between marker genes and their respective biological processes. For example, the marker gene G0S2 in K562 cells is associated with the extrinsic apoptotic signaling pathway. Leukemia cells often exhibit excessive proliferation. In K562 cells, the marker gene G0S2 indirectly promotes cell apoptosis by influencing the cell cycle and inhibiting excessive proliferation. Similarly, lymphocyte cells play a crucial role in the immune system, particularly in antigen processing and presentation. The enrichment results show that the marker genes HLA-DRA, CD74, and HLA-DRB1 in GM12878 cells are closely associated with antigen processing and presentation. These enrichment results further validate the functional significance of the marker genes identified by scWDAC, reflecting the inherent relationships between marker genes in different cell types and their corresponding biological processes.

Enrichment analysis is crucial for elucidating the biological characteristics and functions of transcriptomic data. From the SNARE dataset, the top 20 highest-scoring marker genes are selected for each cell cluster, and gene ontology (GO) annotation analysis is performed using the clusterProfiler tool [44] in R. Fig 5E presents the results of the BP enrichment analysis, highlighting the top 20 biological process (BP) categories sorted by *p*-value. The most significantly enriched processes include dendritic cell antigen processing and presentation, extrinsic apoptotic signaling pathway, humoral immune response, and negative regulation of cytokine production. Furthermore, several processes show moderate enrichment levels, involving regulation of cell growth, ERK1 and ERK2 cascades, positive regulation of the insulin-like growth factor receptor signaling pathway, positive regulation of T-cell activation, and positive regulation of cell-cell adhesion.

Overall, the enrichment analysis indicates that the marker genes identified by scWDAC are closely associated with immune-related biological processes, further validating their functional significance. This further validates the functional significance of these marker genes.

### Ablation analysis

**Fig 6:**
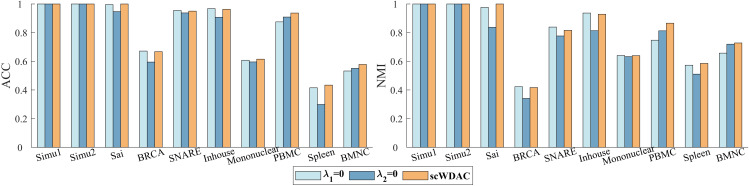
The ablation analysis experiments of scWDAC.

Previous studies have demonstrated that integrating linear and nonlinear information can significantly improve the performance of clustering methods [45,46]. To further validate the effectiveness of this strategy on clustering performance, we conducted an ablation analysis within the scWDAC framework by removing the respective terms, evaluating the contribution of each key component to the overall performance. The impact of component removal on clustering performance is evident from the changes in ACC and NMI, as shown in Fig 6. Specifically, $\lambda_{1}=0$ denotes the removal of the weighted distance penalty term, while $\lambda_{2}=0$ represents the removal of the noise term.

The results showed that the removal of both the weighted distance penalty term and the noise term significantly impacted the model’s performance. For small-scale datasets such as the Sai, BRCA, SNARE, Inhouse, and Mononuclear datasets, the primary challenge here often stems from technical noise and sparsity. Removing the noise term had a more significant impact on the clustering results, with the ACC values decreasing by 5.34%, 10.95%, 1.40%, 5.81%, and 3.19%, respectively. This observation further confirms that effective noise suppression is a prerequisite for robust integration in such scenarios. In contrast, for large-scale datasets with nonlinear features, such as the PBMC and BMNC datasets, these datasets typically contain more complex cellular subpopulations and exhibit stronger nonlinear relationships. Removing the weighted distance penalty term, which is designed to capture such nonlinear and local structures, led to greater performance degradation, with ACC values decreasing by 6.62% and 7.87%, respectively. Overall, the joint adoption of these two components significantly improved the clustering performance of scWDAC across most datasets.

**Fig 7:**
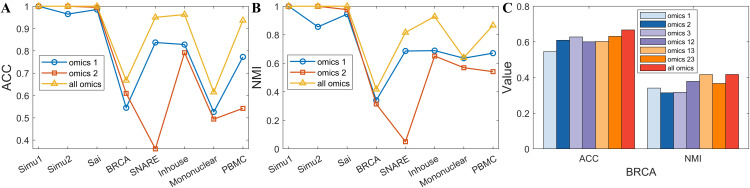
Performance of various combinations of omics on eight test datasets.

To further examine scWDAC’s ability to integrate multi-omics data, we systematically tested all possible combinations of omics layers. As shown in Fig 7, the best performance was consistently achieved only when all omics layers are used simultaneously. In the BRCA dataset, the performance of any pairwise omics layers combination consistently exceeded the weaker omics layer, corroborating the value of cross-omics complementary information. Additionally, the results from the BRCA dataset confirmed that scWDAC can effectively scale to three omics layers.

### Survival analysis on real cancer datasets

We further evaluated scWDAC using two real cancer multi-omics datasets from The Cancer Genome Atlas (TCGA), a comprehensive resource that aggregates molecular profiling and clinical data across multiple cancer types [47]. We selected acute myeloid leukemia (AML) and glioblastoma multiforme (GBM) datasets, which include mRNA expression, DNA methylation, miRNA expression, and clinical annotations. Only samples with all three omics types available were retained. We applied scWDAC to perform sample clustering on these paired multi-omics datasets. Since the number of sample clusters was unknown a priori, we determined the optimal number using the eigengap method based on the learned similarity matrix and obtain the final sample groupings by using spectral algorithm. We then identified the top 10 marker genes for each resulting cluster using the COSG R package. Finally, survival analysis is conducted on samples with available clinical information. Only genes achieving statistical significance (p < 0.05) are retained as prognostic markers. The identified marker genes are integrated with the clinical data to further assess the relationship between these marker genes and patient survival time.

**Fig 8:**
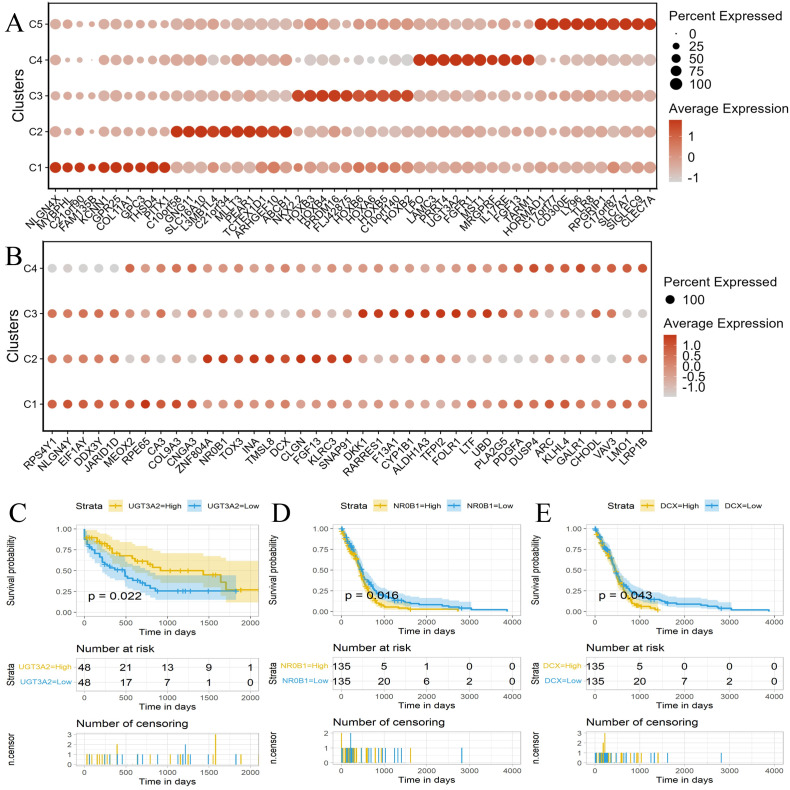
Marker genes identified by scWDAC and their association with patient survival. (A-B) Bubble plots of marker genes identified by scWDAC in the (A) AML and (B) GBM datasets. (C-E) Kaplan–Meier survival curves comparing high-versus low-expression groups of selected marker genes in the (C) AML dataset (UGT3A2 gene), and the GBM dataset for (D) NROB1 and (E) DCX genes.

Fig 8 summarizes marker gene identification and survival analysis for the AML and GBM datasets. Dot plots illustrating marker gene identification for AML and GBM are shown in Fig 8A and 8B, respectively. Marker genes in the AML dataset exhibited more distinct expression patterns and stronger cluster specificity compared to those in GBM. For survival analysis, samples were stratified into “High” and “Low” expression groups based on whether the expression level of each marker gene was above or below the median across samples. In the AML dataset (Fig 8C), high expression of the UGT3A2 was significantly associated with improved survival outcomes (*p* = 0.022). One patient in the high-expression group survived beyond 2,000 days, whereas all patients in the low-expression group experienced the endpoint event. In contrast, for the GBM dataset (Fig 8D and 8E), low expression of NROB1 and DCX was associated with better survival. Two patients in the low-expression groups survived up to 2,000 days, while none in the corresponding high-expression groups reached this time point. These findings highlight the ability of scWDAC to identify marker genes with potential prognostic relevance in cancer, supporting its utility for precision medicine–oriented bioinformatic analyses.

### Computational complexity and convergence analysis

The optimization algorithm involves iterative steps, with its computational complexity dominated by several key matrix operations. Below we detail the most computationally demanding steps among the ten main steps listed in Algorithm 1 (see Section 1 of the S1 File). First, updating the consensus matrix $C^{*}$ is dominated by the SVD operation of an *n*-order square matrix, with computational complexity of *O*(*n*^3^). Similarly, updating the matrices L and R across all views each requires *O*(*Vn*^3^) operations. The update of E can be computed via a closed-form solution, with a complexity of $O(k_{v}n^{2}+k_{v}n)$ for each view, where *k*_*v*_ represents the number of eigenvectors for the *v*-th view. For datasets with *d*_*v*_ > *n*, the Woodbury formula [48] is not required. Consequently, the updates for Z and C across all views, which involve matrix inversion, also scale as *O*(*Vn*^3^). The final step involves performing SVD on $Z^{*}$ and applying spectral clustering to W, with complexity of $O(n^{3}+n^{2})$. Therefore, the per-iteration computational complexity of scWDAC is $O((4V+2)n^{3}+(Vk_{\text{max}}+1)n^{2}+Vk_{\text{max}}n)$, where $k_{\text{max}}=\max\left\{k_{1},\ldots k_{V}\right\}$. Given *t* iterations, the overall computational complexity becomes $O((t(4V+1)+1)n^{3}+(tVk_{\text{max}}+1)n^{2}+tVk_{\text{max}}n)$.

The computational performance of the methods is evaluated by comparing their execution times across ten datasets (Table 1). The “/” symbol indicates instances where results could not be obtained within the specified time (60 hours) or due to memory limitations. The bold fonts indicate the fastest result for each dataset. Among these methods, BREMSC exhibits the longest computation times for most datasets. In contrast, LIGER demonstrates the shortest computation times across most datasets, with a significant advantage for larger datasets. scWDAC performs efficiently on relatively small datasets but became progressively more time‑consuming as the data size increased.

**Table 1:** Computational time (in seconds) of each method on different datasets.

Datasets	Simu1(200)	Simu2(200)	Sai(206)	BRCA(875)	SNARE(1047)	Inhouse(1182)	Mononuclear(2714)	PBMC(6661)	Spleen(16828)	BMNC(30672)
RC_MSC	2.56 ± 0.03	2.72 ± 0.04	69.04 ± 0.75	**9.60 ± 0.35**	34.10 ± 0.27	12.51 ± 0.09	196.51 ± 20.71	1240.17 ± 73.72	28682.50 ± 159.83	49596.98 ± 3150.06
OMVCDR	10.79 ± 0.26	4.64 ± 0.14	93.27 ± 8.05	35.15 ± 0.21	101.75 ± 6.35	110.54 ± 7.76	252.85 ± 38.45	880.32 ± 7.23	3358.18 ± 107.16	11189.17 ± 1129.12
MOSL	2.59 ± 0.06	2.07 ± 0.04	17.31 ± 0.16	40.80 ± 0.42	43.90 ± 0.17	46.88 ± 0.07	437.59 ± 46.76	6548.71 ± 177.46	30684.4 ± 2994.98	167913.77 ± 14446.57
JSNMF	4.06 ± 0.83	2.61 ± 0.72	102.80 ± 2.17	22.51 ± 0.51	39.80 ± 3.14	52.59 ± 2.88	243.44 ± 2.68	1412.67 ± 37.58	4757.66 ± 398.43	/
scAI	0.98 ± 0.11	**0.75 ± 0.01**	71.48 ± 0.64	23.34 ± 1.60	21.91 ± 0.90	29.92 ± 0.16	239.03 ± 6.07	1194.57 ± 43.78	11137.53 ± 221.09	70332.94 ± 3483.84
BREMSC	1379.30 ± 87.93	1996.45 ± 117.13	2110.84 ± 217.33	4297.49 ± 187.64	6091.86 ± 223.52	6873.15 ± 276.54	31884.55 ± 1347.22	24502.85 ± 902.36	/	/
MALS	4.71 ± 0.53	4.52 ± 0.05	62.09 ± 0.71	42.86 ± 0.59	57.30 ± 5.97	51.60 ± 7.51	419.95 ± 32.61	3805.39 ± 9.05	23692.84 ± 725.09	103766.52 ± 4032.32
MVCLRS	1.77 ± 0.82	1.60 ± 0.47	**4.15 ± 0.43**	23.02 ± 0.95	19.27 ± 0.14	39.09 ± 2.94	201.54 ± 19.77	1394.46 ± 77.73	17411.31 ± 475.21	85175.62 ± 2130.25
Seurat	20.16 ± 1.05	12.69 ± 0.87	94.39 ± 2.63	2233.48 ± 91.67	47.42 ± 6.79	36.97 ± 2.10	981.98 ± 50.88	676.37 ± 54.22	3171.80 ± 86.23	2716.21 ± 124.03
LIGER	8.56 ± 0.74	7.03 ± 1.03	130.51 ± 8.91	27.50 ± 1.07	**15.61 ± 0.55**	**11.33 ± 0.48**	**43.45 ± 2.13**	**462.31 ± 27.76**	**531.67 ± 24.63**	**623.32 ± 28.83**
scWDAC	**0.81 ± 0.06**	0.93 ± 0.02	55.85 ± 4.71	14.27 ± 0.48	28.24 ± 1.37	30.06 ± 0.22	401.69 ± 3.42	12669.75 ± 750.15	67156.32 ± 550.44	78287.94 ± 769.81

Owing to the complexity of scWDAC, which involves multiple blocks, it is impractical to prove its theoretical convergence. To numerically assess the convergence of scWDAC, we present the objective value and clustering metrics versus iteration number in S9 Fig on page 12.

## Discussion

The advent of single-cell multi-omics technologies has revolutionized the field of cellular biology by enabling the simultaneous interrogation of genomic, transcriptomic, proteomic, and epigenetic layers within individual cells. These advancements offer unparalleled opportunities to dissect cellular heterogeneity and regulatory networks but also pose significant computational challenges, including high-dimensional noise, modality-specific biases, and the need for effective integrative analysis across disparate data types.

To address these challenges, we propose scWDAC, a novel clustering framework that integrates multi-omics data using weighted distance penalties and adaptive graph regularization. The weighted distance penalty is designed to capture cell-to-cell similarities across different omics modalities, aligning information from various layers in a biologically meaningful manner. This approach reduces the risks associated with low-rank approximations while ensuring cross-modal consistency, thereby significantly improves the robustness of clustering results. A major strength of scWDAC lies in its ability to optimize both global consistency and local accuracy, which are critical for uncovering the complex structures that define cellular heterogeneity. This dual optimization guarantees that the resulting clusters are consistent across the entire dataset while also accurately reflecting the fine-grained differences between individual cells. Extensive experimental validation across ten distinct single-cell multi-omics datasets has demonstrated the superiority of scWDAC compared to existing clustering methods. Notably, scWDAC excels in clustering accuracy, robustness to noise, and biological interpretability, characteristics that are especially important given the high variability and technical noise often found in single-cell multi-omics data. Furthermore, when applied to bulk multi-omics cancer datasets, scWDAC successfully identified marker genes with potential prognostic relevance, underscoring its potential for precision medicine biomarker discovery.

Despite these promising results, the scalability of scWDAC to larger and more complex datasets remains a critical challenge. As the size and complexity of single-cell multi-omics datasets continue to grow, scWDAC’s computational demands may increase. While the current implementation performs well on publicly available datasets, future research should focus on optimizing computational strategies, including exploring parallelization techniques and developing more efficient algorithms. These improvements are essential to ensure that scWDAC can handle the increasing volume of data and maintain performance in large-scale single-cell analyses.

In conclusion, scWDAC offers a powerful tool for integrating multi-omics data to uncover cellular heterogeneity and functional mechanisms. Its application has broad potential in biomedical research, particularly in understanding complex biological processes and identifying cell types. However, its ability to scale to larger datasets and handle the growing demands of large-scale studies will be crucial for its continued success. Future research should focus on optimizing computational efficiency will be crucial for extending the capabilities of scWDAC to meet the challenges of increasingly complex datasets.

## Methods and data

### Model of scWDAC

LRR is a self-representation method that utilizes the data matrix as a dictionary, effectively capturing the global structure of the data [49]. It assumes that data points are sampled from independent subspaces, with points within the same subspace being linearly representable by one another. For a single-omics dataset $X=\left[x_{1},x_{2},\ldots ,x_{n}\right]\in {\mathbb{R}}^{m\times n}$, where *m* and *n* represent the number of features and single cells, respectively, the general LRR model is expressed as


$$\min_{Z,E}\mathrm{rank}(Z)+\lambda {\left\|E\right\|}_{2,1}\text{ s.t. }X=XZ+E,$$ (1)

where $E\in {\mathbb{R}}^{m\times n}$ is a sparse noise matrix that fit the noise, λ>0 is a regularization parameter, and *l*_2,1_ norm encourages row sparsity in E. $Z\in {\mathbb{R}}^{n\times n}$ is a low-rank representation matrix obtained in the latent subspace. Specifica*ll*y, the true segmentation of the data can be revealed by minimizing the rank of Z [50]. However, due to the discrete nature of the rank function, obtaining a solution is challenging. Therefore, the nuclear norm is widely adopted as its convex relaxation, and the optimization problem (1) is reformulated as


$$\min_{Z,E}{\left\|Z\right\|}_{*}+\lambda {\left\|E\right\|}_{2,1}\text{ s.t. }X=XZ+E.$$ (2)

Multi-omics profiles provide complementary information on the same set of cells, enabling a more comprehensive understanding of cellular behavior [20]. To leverage the complementary nature of multi-omics data, LRR-based methods have been extended to a multi-omics framework. Given a multi-omics data $X=\{X^{1},X^{2},\ldots X^{V}\}$, where $X^{v}\in {\mathbb{R}}^{m_{v}\times n}$ represents the feature matrix for the *v*-th omics, with *m*_*v*_ and *n* denoting the number of features and the number of cells, respectively. The extended form of LRR in multi-omics is formulated as


$$\min_{Z^{v},E^{v}}\sum_{v=1}^{V}({\left\|Z^{v}\right\|}_{*}+\lambda {\left\|E^{v}\right\|}_{2,1})\text{ s.t. }X^{v}=X^{v}Z^{v}+E^{v}.$$ (3)

where $Z^{v}\in {\mathbb{R}}^{n\times n}$ and $E^{v}\in {\mathbb{R}}^{m_{v}\times n}$ represent the view-specific representation matrix and the noise matrix for the *v*-th omics layer, respectively.

Although LRR recovers the global information of the cells, it ignores inherent local structure information in the data. To incorporate local geometry, the weighted regularization term $\sum_{i,j}{\left\|x_{i}-x_{j}\right\|}_{2}^{2}z_{ij}$ has been widely adopted [51]. This term, however, only characterizes linear relationships between cells, whereas gene-regulatory programs and cell–cell communication exhibit pronounced nonlinearities. To capture these nonlinear local dependencies, we introduce a weight matrix P that adaptively penalizes inter-cell distances.


$$\begin{array}{c} p_{ij}=\left\{ \begin{array}{cc} e^{-\frac{{\left\|x_{i}-x_{j}\right\|}_{2}^{2}}{2\sigma^{2}}}, & x_{i}\in \text{KNN}\left(x_{j}\right)\\ 1, & \text{otherwise.} \end{array}\right. \end{array}$$ (4)

where $\text{KNN}(x_{j})$ denotes the set of *k*-nearest neighbors (KNN) of cell *j*, with the number of neighbors *k* and σ set to 10 and 1, respectively. Therefore, the weighted distance penalty regularization term is $\sum_{i,j}p_{ij}{\left\|x_{i}-x_{j}\right\|}_{2}^{2}z_{ij}$. When cells *i* and *j* are mutual KNNs, both ${\left\|x_{i}-x_{j}\right\|}_{2}^{2}$ and the penalty weight *p*_*ij*_ (*p*_*ij*_ < 1) are small. As a result, the model tends to learn larger scores *z*_*ij*_, which increases the likelihood of the two cells being assigned to the same cell cluster. Furthermore, the diagonal elements are set to zero to prevent cells from representing themselves, and each row is constrained to sum to one. With the introduction of the weighted distance penalty regularization term, the optimization problem (3) is reformulated as


$$\begin{array}{c} \min_{Z^{v},E^{v}}\sum_{v=1}^{V}({\left\|Z^{v}\right\|}_{*}+\lambda_{1}\sum_{i,j}p_{ij}^{v}{\left\|x_{i}^{v}-x_{j}^{v}\right\|}_{2}^{2}z_{ij}^{v}+\lambda_{2}{\left\|E^{v}\right\|}_{2,1})\\ \text{ s.t. }X^{v}=X^{v}Z^{v}+E^{v},\mathrm{diag}(Z^{v})=\text{0},z_{ij}^{v}\geq 0,\sum_{j}z_{ij}^{v}=1. \end{array}$$ (5)

The key to spectral clustering lies in constructing a high-quality representation matrix. Directly applying the representation matrix may capture noise and outliers, leading to inaccurate models or poor generalization. In addition to complementarity, consistency is equally crucial for enhancing clustering performance in multi-omics analysis. Consistency refers to the common features across different omics, specifically, the shared representation structure [52]. Inspired by the consistency strategy introduced in RC_MSC [53], the representation matrix $Z^{v}$ is decomposed into three matrices $L^{v}$, C, and ${R^{v}}^{T}$. The model in (5) is then optimized as follows:


$$\begin{array}{c} \min_{Z^{v},L^{v},C,R^{v},E^{v}}{\left\|C\right\|}_{*}+\sum_{v=1}^{V}(\lambda_{1}\sum_{i,j}p_{ij}^{v}{\left\|x_{i}^{v}-x_{j}^{v}\right\|}_{2}^{2}z_{ij}^{v}+\lambda_{2}{\left\|E^{v}\right\|}_{2,1})\\ \text{s.t. }X^{v}=X^{v}Z^{v}+E^{v},Z^{v}=L^{v}C{R^{v}}^{T},{L^{v}}^{T}L^{v}=I,{R^{v}}^{T}R^{v}=I,\\ \;\mathrm{diag}(Z^{v})=\text{0},z_{ij}^{v}\geq 0,\sum_{j}z_{ij}^{v}=1, \end{array}$$ (6)

where $C\in {\mathbb{R}}^{n\times n}$ is the consensus representation matrix of $Z^{v}$ across all omics, designed to preserve key features and promote consistency across omics. The left factor matrix $L^{v}\in {\mathbb{R}}^{n\times n}$ and the right factor matrix $R^{v}\in {\mathbb{R}}^{n\times n}$ represent the basis vectors of $Z^{v}$ along the two extended directions, respectively. Finally, the orthogonality constraint is imposed to prevent trivial solutions.

Owing to inherent noise and biases in single-cell sequencing technologies, the reliability of omics data varies. To mitigate the impact of noise and improve multi-omics integration, we adopt an adaptive consistency graph regularization strategy that assigns distinct weights to each omics layer. Taking these factors into account, the final optimization problem for scWDAC is formulated as follows:


$$\begin{array}{c} \min_{Z^{v},C^{*},L^{v},C^{v},R^{v},E^{v},w^{v}}{\left\|C^{*}\right\|}_{*}+\sum_{v=1}^{V}(\lambda_{1}\sum_{i,j}p_{ij}^{v}{\left\|x_{i}^{v}-x_{j}^{v}\right\|}_{2}^{2}z_{ij}^{v}\\ \;+w^{v}{\left\|C^{v}-C^{*}\right\|}_{F}^{2}+\lambda_{2}{\left\|E^{v}\right\|}_{2,1})\\ \text{s.t. }X^{v}=X^{v}Z^{v}+E^{v},Z^{v}=L^{v}C^{v}{R^{v}}^{T},{L^{v}}^{T}L^{v}=I,{R^{v}}^{T}R^{v}=I,\\ \;\mathrm{diag}(Z^{v})=\text{0},z_{ij}^{v}\geq 0,\sum_{j}z_{ij}^{v}=1, \end{array}$$ (7)

where $C^{*}\in {\mathbb{R}}^{n\times n}$ is the consensus representation matrix, and *w*^v^ denotes the weight of the *v*-th omics in the adaptive graph regularization term. The term $\sum_{v=1}^{V}w^{v}{\left\|C^{v}-C^{*}\right\|}_{F}^{2}$ enforces cross-omics consistency while capturing underlying shared structure.

The detailed optimization process for each variable is provided in the Section 1 of S1 File. It should be noted that $C^{v}$ serves as an intermediate representation that coordinates shared information, allowing the model to capture both consistency and complementarity across modalities. The iterative optimization process refines both $C^{v}$ and $Z^{v}$, ensuring that the final outputs of $Z^{v}$ accurately capture the unique features of each modality while preserving the shared structure.

Based on the obtained representation matrix $Z^{v}$ for each omics data, and the similarity matrix $Z^{*}$ is given by $Z^{*}=\sum_{v=1}^{V}Z^{v}$. However, the similarity matrix $Z^{*}$ obtained through this fusion strategy contains a significant amount of redundant information, which severely hampers the performance of spectral clustering. The values in the similarity matrix represent the importance of the corresponding intrinsic structural information. Specifically, for each column of $Z^{*}$, the elements are sorted in descending order, and the cumulative sum is computed until reaching τ% of the total sum. The selected elements are retained, while the others are discarded. In general, larger sample sizes correspond to more irrelevant information, so the information retention rate is inversely proportional to τ and the sample size *n*, defined as


$$\tau =\varepsilon_{1}+\frac{1}{\varepsilon_{2}n+\varepsilon_{3}},$$ (8)

where τ is the information retention ratio, and $\varepsilon_{1}$, $\varepsilon_{2}$, and $\varepsilon_{3}$ are invariant constants with consistent values across diverse datasets.

Consider the similarity matrix $Z^{*}$ based on low-rank representations, where the principal component information between any two vectors from the same subspace is greater than that between vectors derived from different subspaces [54]. Specifically, we perform the skinny singular value decomposition of $Z^{*}$: $Z^{*}=U𝛬V^{T}$. We then construct the angle information matrix $M=U\sqrt{𝛬}$ and define the similarity matrix as


$$w_{ij}={\left(\frac{m_{i}^{T}m_{j}}{{\left\|m_{i}\right\|}_{2}{\left\|m_{j}\right\|}_{2}}\right)}^{k},$$ (9)

where $m_{i}$ and $m_{j}$ represent the *i*-th and *j*-th rows of M, respectively. Furthermore, the term *k* = 2 ensures that all values in W for subspace clustering are positive [55]. Finally, the similarity matrix W is employed for spectral clustering. Algorithm 1 outlines the complete steps of scWDAC in Section 1 of the S1 File.

### Data description

In this paper, we utilize ten paired single-cell multi-omics datasets with accurate cell type annotations, which have been previously employed in academic studies to assess model efficacy. The details of these datasets are summarized in Table 2.

**Table 2:** Details of the benchmark datasets.

Datasets	Cells	Type of features	Number of features	Clusters
Simu1 [24]	200	scRNA/scATAC	1,000/5,000	3
Simu2 [24]	200	scRNA/scATAC	1,000/5,000	3
Sai[37]	206	scRNA/scATAC	49,073/207,202	3
BRCA^1^	875	mRNA/DNA methylation/miRNA	1,000/1,000/503	5
SNARE [56]	1,047	scRNA/scATAC	500/7,136	4
Inhouse [33]	1,182	scRNA/ADT	1,000/10	6
Mononuclear [30]	2,714	scRNA/scATAC	2,000/5,000	9
PBMC [31]	6,661	scRNA/ADT	33,538/14	7
Spleen [57]	16,828	scRNA/ADT	13,553/112	35
BMNC [58]	30,672	scRNA/scATAC	1,000/25	27

^1^https://gdac.broadinstitute.org/.

## Supporting information

S1 File Supplementary notes for scWDAC. 1. Details of the optimization processes. 2. Evaluation metrics. 3. Numerical results of ACC and NMI. 4. Visualization of clustering results. 5. Parameter sensitivity analysis. 6. Comparison of marker gene identification. 7. Convergence analysis.
(PDF)

S1 Fig Visualization of the clustering results. (TIF)

S2 Fig Comparison of heatmaps of similarity matrices from different methods. (TIF)

S3 Fig Clustering ACC and NMI with respect to the parameters and across various datasets. (TIF)

S4 Fig Exploring the impact of KNN neighborhood size on model performance. (TIF)

S5 Fig Comparison of ACC and NMI across eight datasets under various kernel functions. (TIF)

S6 Fig The UMAP Visualization based on four different label conditions: True (A), scWDAC (B), JSNMF (C), and scAI (D). (TIF)

S7 Fig Bubble plots of marker genes identified by truth labels (A), scWDAC (B), JSNMF (C), and scAI (D). (TIF)

S8 Fig Enrichment analysis of predicted marker genes: True markers (A), scWDAC (B), JSNMF (C), scAI (D). (TIF)

S9 Fig (A). The convergence curves of scWDAC on all datasets. (B). ACC and NMI versus the iteration number on the corresponding datasets. (TIF)

S1 Table The numerical comparison of ACC (mean% ± standard%). (XLSX)

S2 Table The numerical comparison of NMI (mean% ± standard%). (XLSX)

S3 Table Evaluation of KNN neighbors (*k* = 10) in embedding space. (XLSX)

S4 Table Evaluation of KNN neighbors (*k* = 20) in embedding space. (XLSX)

S5 Table Evaluation of KNN neighbors (*k* = 30) in embedding space. (XLSX)

S6 Table Comparison of the overlap between predicted marker genes from three methods and true marker genes. (XLSX)

## References

[1] Lawson DA, Kessenbrock K, Davis RT, Pervolarakis N, Werb Z. Tumour heterogeneity and metastasis at single-cell resolution. Nat Cell Biol. 2018;20(12):1349–60. doi: 10.1038/s41556-018-0236-7 30482943 PMC6477686

[2] Patel AP, Tirosh I, Trombetta JJ, Shalek AK, Gillespie SM, Wakimoto H, et al. Single-cell RNA-seq highlights intratumoral heterogeneity in primary glioblastoma. Science. 2014;344(6190):1396–401. doi: 10.1126/science.1254257 24925914 PMC4123637

[3] Potter SS. Single-cell RNA sequencing for the study of development, physiology and disease. Nat Rev Nephrol. 2018;14(8):479–92. doi: 10.1038/s41581-018-0021-7 29789704 PMC6070143

[4] Macaulay IC, Ponting CP, Voet T. Single-Cell Multiomics: Multiple Measurements from Single Cells. Trends Genet. 2017;33(2):155–68. doi: 10.1016/j.tig.2016.12.003 28089370 PMC5303816

[5] Buenrostro JD, Wu B, Chang HY, Greenleaf WJ. ATAC-seq: A Method for Assaying Chromatin Accessibility Genome-Wide. Curr Protoc Mol Biol. 2015;109:21.29.1-21.29.9. doi: 10.1002/0471142727.mb2129s109 25559105 PMC4374986

[6] Khan R, Mallory X. Assessing the performance of methods for cell clustering from single-cell DNA sequencing data. PLoS Comput Biol. 2023;19(10):e1010480. doi: 10.1371/journal.pcbi.1010480 37824596 PMC10597505

[7] Cao J, Cusanovich DA, Ramani V, Aghamirzaie D, Pliner HA, Hill AJ, et al. Joint profiling of chromatin accessibility and gene expression in thousands of single cells. Science. 2018;361(6409):1380–5. doi: 10.1126/science.aau0730 30166440 PMC6571013

[8] Kiselev VY, Andrews TS, Hemberg M. Challenges in unsupervised clustering of single-cell RNA-seq data. Nat Rev Genet. 2019;20(5):273–82. doi: 10.1038/s41576-018-0088-9 30617341

[9] Sun N, Yu X, Li F, Liu D, Suo S, Chen W, et al. Inference of differentiation time for single cell transcriptomes using cell population reference data. Nat Commun. 2017;8(1):1856. doi: 10.1038/s41467-017-01860-2 29187729 PMC5707349

[10] Papalexi E, Satija R. Single-cell RNA sequencing to explore immune cell heterogeneity. Nat Rev Immunol. 2018;18(1):35–45. doi: 10.1038/nri.2017.76 28787399

[11] Kiselev VY, Kirschner K, Schaub MT, Andrews T, Yiu A, Chandra T, et al. SC3: consensus clustering of single-cell RNA-seq data. Nat Methods. 2017;14(5):483–6. doi: 10.1038/nmeth.4236 28346451 PMC5410170

[12] Wang B, Zhu J, Pierson E, Ramazzotti D, Batzoglou S. Visualization and analysis of single-cell RNA-seq data by kernel-based similarity learning. Nat Methods. 2017;14(4):414–6. doi: 10.1038/nmeth.4207 28263960

[13] Zheng R, Li M, Liang Z, Wu F-X, Pan Y, Wang J. SinNLRR: a robust subspace clustering method for cell type detection by non-negative and low-rank representation. Bioinformatics. 2019;35(19):3642–50. doi: 10.1093/bioinformatics/btz139 30821315

[14] Zhang W, Li Y, Zou X. SCCLRR: A Robust Computational Method for Accurate Clustering Single Cell RNA-Seq Data. IEEE J Biomed Health Inform. 2021;25(1):247–56. doi: 10.1109/JBHI.2020.2991172 32356764

[15] Wu W, Ma X. Joint learning dimension reduction and clustering of single-cell RNA-sequencing data. Bioinformatics. 2020;36(12):3825–32. doi: 10.1093/bioinformatics/btaa231 32246821

[16] Zhang W, Xue X, Zheng X, Fan Z. NMFLRR: Clustering scRNA-Seq Data by Integrating Nonnegative Matrix Factorization With Low Rank Representation. IEEE J Biomed Health Inform. 2022;26(3):1394–405. doi: 10.1109/JBHI.2021.3099127 34310328

[17] Zhang N-N, Liu J-X, Zheng C-H, Wang J. SLRRSC: Single-Cell Type Recognition Method Based on Similarity and Graph Regularization Constraints. IEEE J Biomed Health Inform. 2022;26(7):3556–66. doi: 10.1109/JBHI.2022.3148286 35120014

[18] Cheng Y, Ma X. scGAC: a graph attentional architecture for clustering single-cell RNA-seq data. Bioinformatics. 2022;38(8):2187–93. doi: 10.1093/bioinformatics/btac099 35176138

[19] Zhang W, Xu Y, Zheng X, Shen J, Li Y. Identifying cell types by lasso-constraint regularized Gaussian graphical model based on weighted distance penalty. Brief Bioinform. 2024;25(6):bbae572. doi: 10.1093/bib/bbae572 39541187 PMC11562834

[20] Peng A, Mao X, Zhong J, Fan S, Hu Y. Single-Cell Multi-Omics and Its Prospective Application in Cancer Biology. Proteomics. 2020;20(13):e1900271. doi: 10.1002/pmic.201900271 32223079

[21] Gohil SH, Iorgulescu JB, Braun DA, Keskin DB, Livak KJ. Applying high-dimensional single-cell technologies to the analysis of cancer immunotherapy. Nat Rev Clin Oncol. 2021;18(4):244–56. doi: 10.1038/s41571-020-00449-x 33277626 PMC8415132

[22] Cao K, Bai X, Hong Y, Wan L. Unsupervised topological alignment for single-cell multi-omics integration. Bioinformatics. 2020;36(Suppl_1):i48–56. doi: 10.1093/bioinformatics/btaa443 32657382 PMC7355262

[23] Wang X, Sun Z, Zhang Y, Xu Z, Xin H, Huang H, et al. BREM-SC: a bayesian random effects mixture model for joint clustering single cell multi-omics data. Nucleic Acids Res. 2020;48(11):5814–24. doi: 10.1093/nar/gkaa314 32379315 PMC7293045

[24] Jin S, Zhang L, Nie Q. scAI: an unsupervised approach for the integrative analysis of parallel single-cell transcriptomic and epigenomic profiles. Genome Biol. 2020;21(1):25. doi: 10.1186/s13059-020-1932-8 32014031 PMC6996200

[25] Argelaguet R, Arnol D, Bredikhin D, Deloro Y, Velten B, Marioni JC, et al. MOFA+: a statistical framework for comprehensive integration of multi-modal single-cell data. Genome Biol. 2020;21(1):111. doi: 10.1186/s13059-020-02015-1 32393329 PMC7212577

[26] Zhanpeng H, Jiekang W. A Multiview Clustering Method With Low-Rank and Sparsity Constraints for Cancer Subtyping. IEEE/ACM Trans Comput Biol Bioinform. 2022;19(6):3213–23. doi: 10.1109/TCBB.2021.3122917 34705654

[27] Liu H, Shang M, Zhang H, Liang C. Cancer Subtype Identification based on Multi-view Subspace Clustering with Adaptive Local Structure Learning. In: 2021 IEEE International Conference on Bioinformatics and Biomedicine (BIBM), 2021. 484–90. 10.1109/bibm52615.2021.9669659

[28] Ma Y, Sun Z, Zeng P, Zhang W, Lin Z. JSNMF enables effective and accurate integrative analysis of single-cell multiomics data. Brief Bioinform. 2022;23(3):bbac105. doi: 10.1093/bib/bbac105 35380624

[29] Eltager M, Abdelaal T, Mahfouz A, Reinders MJT. scMoC: single-cell multi-omics clustering. Bioinform Adv. 2022;2(1):vbac011. doi: 10.1093/bioadv/vbac011 36699396 PMC9710707

[30] Zeng P, Ma Y, Lin Z. scAWMV: an adaptively weighted multi-view learning framework for the integrative analysis of parallel scRNA-seq and scATAC-seq data. Bioinformatics. 2023;39(1):btac739. doi: 10.1093/bioinformatics/btac739 36383176 PMC9805575

[31] Jiang H, Zhan S, Ching W-K, Chen L. Robust joint clustering of multi-omics single-cell data via multi-modal high-order neighborhood Laplacian matrix optimization. Bioinformatics. 2023;39(7):btad414. doi: 10.1093/bioinformatics/btad414 37382572 PMC10329495

[32] Qiu Y, Guo D, Zhao P, Zou Q. scMNMF: a novel method for single-cell multi-omics clustering based on matrix factorization. Brief Bioinform. 2024;25(3):bbae228. doi: 10.1093/bib/bbae228 38754408 PMC11097994

[33] Chen F, Zou G, Wu Y, Ou-Yang L. Clustering single-cell multi-omics data via graph regularized multi-view ensemble learning. Bioinformatics. 2024;40(4):btae169. doi: 10.1093/bioinformatics/btae169 38547401 PMC11015955

[34] Stuart T, Butler A, Hoffman P, Hafemeister C, Papalexi E, Mauck WM 3rd, et al. Comprehensive Integration of Single-Cell Data. Cell. 2019;177(7):1888-1902.e21. doi: 10.1016/j.cell.2019.05.031 31178118 PMC6687398

[35] Cao K, Gong Q, Hong Y, Wan L. A unified computational framework for single-cell data integration with optimal transport. Nat Commun. 2022;13(1):7419. doi: 10.1038/s41467-022-35094-8 36456571 PMC9715710

[36] Bai X, Duren Z, Wan L, Xia LC. Joint inference of clonal structure using single-cell genome and transcriptome sequencing data. NAR Genom Bioinform. 2024;6(1):lqae017. doi: 10.1093/nargab/lqae017 38486887 PMC10939367

[37] Wang H, Liu Z, Ma X. Learning Consistency and Specificity of Cells From Single-Cell Multi-Omic Data. IEEE J Biomed Health Inform. 2024;28(5):3134–45. doi: 10.1109/JBHI.2024.3370868 38709615

[38] Cai D, He X, Han J. Document clustering using locality preserving indexing. IEEE Trans Knowl Data Eng. 2005;17(12):1624–37. doi: 10.1109/tkde.2005.198

[39] Strehl A, Ghosh J. Cluster ensembles—a knowledge reuse framework for combining multiple partitions. J Mach Learn Res. 2002;3:583–617. doi: 10.1162/153244303321897735

[40] Dai M, Pei X, Wang X-J. Accurate and fast cell marker gene identification with COSG. Brief Bioinform. 2022;23(2):bbab579. doi: 10.1093/bib/bbab579 35048116

[41] Bullinger L, Schlenk RF, Götz M, Botzenhardt U, Hofmann S, Russ AC, et al. PRAME-induced inhibition of retinoic acid receptor signaling-mediated differentiation--a possible target for ATRA response in AML without t(15;17). Clin Cancer Res. 2013;19(9):2562–71. doi: 10.1158/1078-0432.CCR-11-2524 23444226

[42] Xie W, Liu W, Wang L, Li S, Liao Z, Xu H, et al. Embryonic stem cell related gene regulates alternative splicing of transcription factor 3 to maintain human embryonic stem cells’ self-renewal and pluripotency. Stem Cells. 2024;42(6):540–53. doi: 10.1093/stmcls/sxae020 38393342

[43] Zhang X, Lan Y, Xu J, Quan F, Zhao E, Deng C, et al. CellMarker: a manually curated resource of cell markers in human and mouse. Nucleic Acids Res. 2019;47(D1):D721–8. doi: 10.1093/nar/gky900 30289549 PMC6323899

[44] Wu T, Hu E, Xu S, Chen M, Guo P, Dai Z, et al. Clusterprofiler 4.0: A universal enrichment tool for interpreting omics data. Innovation. 2021;2(3):100141. doi: 10.1016/j.xinn.2021.100141 34557778 PMC8454663

[45] Dizaji KG, Herandi A, Deng C, Cai W, Huang H. Deep Clustering via Joint Convolutional Autoencoder Embedding and Relative Entropy Minimization. In: 2017 IEEE International Conference on Computer Vision (ICCV), 2017. 5747–56. 10.1109/iccv.2017.612

[46] Xu J, Ren Y, Tang H, Pu X, Zhu X, Zeng M, et al. Multi-VAE: Learning Disentangled View-common and View-peculiar Visual Representations for Multi-view Clustering. In: 2021 IEEE/CVF International Conference on Computer Vision (ICCV), 2021. 9214–23. 10.1109/iccv48922.2021.00910

[47] Cancer Genome Atlas Research Network, Weinstein JN, Collisson EA, Mills GB, Shaw KRM, Ozenberger BA, et al. The Cancer Genome Atlas Pan-Cancer analysis project. Nat Genet. 2013;45(10):1113–20. doi: 10.1038/ng.2764 24071849 PMC3919969

[48] Riedel KS. A Sherman–Morrison–Woodbury Identity for Rank Augmenting Matrices with Application to Centering. SIAM J Matrix Anal & Appl. 1992;13(2):659–62. doi: 10.1137/0613040

[49] Zhuang L, Gao H, Lin Z, Ma Y, Zhang X, Yu N. Non-negative low rank and sparse graph for semi-supervised learning. In: 2012 IEEE Conference on Computer Vision and Pattern Recognition, 2012. 2328–35. 10.1109/CVPR.2012.6247944

[50] Xu C, Lin Z, Zha H. A unified convex surrogate for the Schatten-p norm. In: Proceedings of the AAAI Conference on Artificial Intelligence, 2017. 10.1609/aaai.v31i1.10646

[51] Fu Z, Zhao Y, Chang D, Wang Y, Wen J. Latent Low-Rank Representation With Weighted Distance Penalty for Clustering. IEEE Trans Cybern. 2023;53(11):6870–82. doi: 10.1109/TCYB.2022.3166545 35507611

[52] Xu C, Tao D, Xu C. A survey on multi-view learning. arXiv preprint. 2013. 10.48550/arXiv.1304.5634

[53] Guo J, Sun Y, Gao J, Hu Y, Yin B. Rank Consistency Induced Multiview Subspace Clustering via Low-Rank Matrix Factorization. IEEE Trans Neural Netw Learn Syst. 2022;33(7):3157–70. doi: 10.1109/TNNLS.2021.3071797 33882005

[54] Chen J, Yang S, Mao H, Fahy C. Multiview Subspace Clustering Using Low-Rank Representation. IEEE Trans Cybern. 2022;52(11):12364–78. doi: 10.1109/TCYB.2021.3087114 34185655

[55] Chen J, Mao H, Sang Y, Yi Z. Subspace clustering using a symmetric low-rank representation. Knowledge-Based Systems. 2017;127:46–57. doi: 10.1016/j.knosys.2017.02.031

[56] Zuo C, Dai H, Chen L. Deep cross-omics cycle attention model for joint analysis of single-cell multi-omics data. Bioinformatics. 2021;37(22):4091–9. doi: 10.1093/bioinformatics/btab403 34028557

[57] Gayoso A, Steier Z, Lopez R, Regier J, Nazor KL, Streets A, et al. Joint probabilistic modeling of single-cell multi-omic data with totalVI. Nat Methods. 2021;18(3):272–82. doi: 10.1038/s41592-020-01050-x 33589839 PMC7954949

[58] Hu D, Liang K, Dong Z, Wang J, Zhao Y, He K. Effective multi-modal clustering method via skip aggregation network for parallel scRNA-seq and scATAC-seq data. Brief Bioinform. 2024;25(2):bbae102. doi: 10.1093/bib/bbae102 38493338 PMC10944573

