# Supplementary material for: Clustering single-cell multi-omics data via weighted distance penalty and adaptive consistent graph regularization
Source: PLoS Comput Biol. 2026 Apr 3;22(4):e1014110. doi: 10.1371/journal.pcbi.1014110 (PMC13048436; doi:10.1371/journal.pcbi.1014110)
Supplement: S1 File — 1. Details of the optimization processes. 2. Evaluation metrics. 3. Numerical results of ACC and NMI. 4. Visualization of clustering results. 5. Parameter sensitivity analysis. 6. Comparison of marker gene identification. 7. Convergence analysis. (PDF) [file pcbi.1014110.s001.pdf]

Supplementary materials of "Clustering Single-Cell Multi-Omics Data via Weighted Distance Penalty and Adaptive Consistent Graph Regularization"

1 Model optimization

Model optimization

Owing to the non-convex nature of the model, obtaining a global optimal solution is infeasible. Therefore, the Alternating Direction Method of Multiplier (ADMM) [1] is utilized to solve the problem. Specifically, the optimization process is simplified by introducing an auxiliary variable $\boldsymbol{S}$, and the augmented Lagrangian function is formulated as follows:

$$
\begin{aligned}
& L\left(\boldsymbol{Z}^v, \boldsymbol{S}^v, \boldsymbol{C}^*, \boldsymbol{L}^v, \boldsymbol{C}^v, \boldsymbol{R}^v, \boldsymbol{E}^v, \boldsymbol{Q}_1^v, \boldsymbol{Q}_2^v, \boldsymbol{Q}_3^v, w^v\right)=\left\|\boldsymbol{C}^*\right\|_*+ \\
& \sum_{v=1}^V\left(\lambda_1 \sum_{i,j} p_{ij}^v\left\|\boldsymbol{x}_i^v-\boldsymbol{x}_j^v\right\|_2^2 s_{ij}^v+w^v\left\|\boldsymbol{C}^v-\boldsymbol{C}^*\right\|_F^2+\lambda_2\left\|\boldsymbol{E}^v\right\|_{2,1}\right) \\
& +\sum_{v=1}^V \frac{\mu}{2}\left(\left\|\boldsymbol{X}^v-\boldsymbol{X}^v\boldsymbol{Z}^v-\boldsymbol{E}^v+\frac{\boldsymbol{Q}_1^v}{\mu}\right\|_F^2+\left\|\boldsymbol{Z}^v-\boldsymbol{L}^v\boldsymbol{C}^v\boldsymbol{R}^{vT}+\frac{\boldsymbol{Q}_2^v}{\mu}\right\|_F^2\right. \\
& \left.+\left\|\boldsymbol{Z}^v-\boldsymbol{S}^v+\frac{\boldsymbol{Q}_3^v}{\mu}\right\|_F^2\right) \\
& \text{s.t. } \boldsymbol{L}^{vT}\boldsymbol{L}^v=\boldsymbol{I}, \boldsymbol{R}^{vT}\boldsymbol{R}^v=\boldsymbol{I}, \operatorname{diag}\left(\boldsymbol{S}^v\right)=\mathbf{0}, s_{ij}^v \geq 0, \sum_j s_{ij}^v=1.
\end{aligned}
\tag{1}
$$

According to ADMM, each variable is optimized while keeping the other variables fixed at their latest values. The update process for each variable is as follows:

Step 1: By fixing other variables, the sub-optimization problem for variable $\boldsymbol{C}^*$ is shown as

$$
\begin{aligned}
& \min_{\boldsymbol{C}^*}\left\|\boldsymbol{C}^*\right\|_*+\sum_{v=1}^V w^v\left\|\boldsymbol{C}^v-\boldsymbol{C}^*\right\|_F^2 \\
& =\left\|\boldsymbol{C}^*\right\|_*+\sum_{v=1}^V w^v \operatorname{Tr}\left(-2\boldsymbol{C}^{vT}\boldsymbol{C}^*\right)+\operatorname{Tr}\left(\boldsymbol{C}^{*T}\boldsymbol{C}^*\right)\sum_{v=1}^V w^v+\boldsymbol{B} \\
& =\left\|\boldsymbol{C}^*\right\|_*+\sum_{v=1}^V w^v\left(\operatorname{Tr}\left(\boldsymbol{C}^{*T}\boldsymbol{C}^*\right)+\frac{\sum_{v=1}^V w^v \operatorname{Tr}\left(-2\boldsymbol{C}^{*T}\boldsymbol{C}^v\right)}{\sum_{v=1}^V w^v}\right)+\boldsymbol{B} \\
& =\left\|\boldsymbol{C}^*\right\|_*+\left\|\boldsymbol{C}^*-\frac{\sum_{v=1}^V w^v\boldsymbol{C}^v}{\sum_{v=1}^V w^v}\right\|_F^2 \times \sum_{v=1}^V w^v+\widetilde{\boldsymbol{B}},
\end{aligned}
\tag{2}
$$

where $\boldsymbol{B}$ and $\widetilde{\boldsymbol{B}}$ are independent of $\boldsymbol{C}^*$. Therefore, the optimization problem (2) can be rewritten as

$$
\min_{\boldsymbol{C}^*} \beta\left\|\boldsymbol{C}^*\right\|_*+\frac{1}{2}\left\|\boldsymbol{C}^*-\widetilde{\boldsymbol{C}}\right\|_F^2,
\tag{3}
$$

where $\widetilde{\boldsymbol{C}} = \frac{\sum_{v=1}^{V} w^v \boldsymbol{C}^v}{\sum_{v=1}^{V} w^v}$ and $\beta = \frac{1}{2\sum_{v=1}^{V} w^v}$. As noted in [2], the above problem can be solved by using the singular value thresholding (SVT) operator. The detailed update rule for $\boldsymbol{C}^*$ is given by

$$\boldsymbol{C}^* = \boldsymbol{U}\varSigma_\beta \boldsymbol{V}^T, \tag{4}$$

where $\varSigma_\beta = \mathrm{diag}[(\varsigma_1 - \beta)_+, \ldots, (\varsigma_r - \beta)_+]$ with $x_+ = \max(x, 0)$, and $\boldsymbol{U}\varSigma\boldsymbol{V}^T$ is the SVD of matrix $\widetilde{\boldsymbol{C}}$ with $\varSigma = \mathrm{diag}[\varsigma_1, \ldots, \varsigma_r]$.

Step 2: Holding all other variables are constant, the variable $\boldsymbol{E}^v$ can be determined by solving the following minimization problem:

$$\min_{\boldsymbol{E}^v} \lambda_2 \left\|\boldsymbol{E}^v\right\|_{2,1} + \frac{\mu}{2}\left\|\boldsymbol{X}^v - \boldsymbol{X}^v\boldsymbol{Z}^v - \boldsymbol{E}^v + \frac{\boldsymbol{Q}_1^v}{\mu}\right\|_F^2. \tag{5}$$

According to [3], the solution for each column $\boldsymbol{E}^v$ can be obtained as follows:

$$\boldsymbol{E}^v_{:,i} = \begin{cases} \frac{\left\|\Theta^v_{:,i}\right\|_2 - \frac{\lambda_2}{\mu}}{\left\|\Theta^v_{:,i}\right\|_2}\Theta^v_{:,i}, & \left\|\Theta^v_{:,i}\right\|_2 > \frac{\lambda_2}{\mu} \\ 0, & \text{otherwise.} \end{cases} \tag{6}$$

where $\Theta^v = \boldsymbol{X}^v - \boldsymbol{X}^v\boldsymbol{Z}^v + \frac{\boldsymbol{Q}_1^v}{\mu}$.

Step 3: Fixing the other variables, the optimization problem for $\boldsymbol{L}^v$ and $\boldsymbol{R}^v$ can be expressed as

$$\min_{\boldsymbol{L}^{vT}\boldsymbol{L}^v = \boldsymbol{I}, \boldsymbol{R}^{vT}\boldsymbol{R}^v = \boldsymbol{I}} \left\|\boldsymbol{Z}^v - \boldsymbol{L}^v\boldsymbol{C}^v\boldsymbol{R}^{vT} + \frac{\boldsymbol{Q}_2^v}{\mu}\right\|_F^2. \tag{7}$$

By keeping the $\boldsymbol{R}^v$ as constant, the above equation can be rewritten as

$$\min_{\boldsymbol{L}^{vT}\boldsymbol{L}^v = \boldsymbol{I}} \left\|\boldsymbol{L}^v - \widetilde{\boldsymbol{L}}^v\right\|_F^2, \tag{8}$$

where $\widetilde{\boldsymbol{L}}^v = \left(\boldsymbol{Z}^v + \frac{\boldsymbol{Q}_2^v}{\mu}\right)\boldsymbol{R}^v\boldsymbol{C}^{vT}$. The above equation is the classical orthogonal procrustes problem. Based on the results obtained in [4], the closed-form solution for $\boldsymbol{L}^v$ is given by $\boldsymbol{L}^v = \boldsymbol{A}^v\boldsymbol{B}^{vT}$, where $\boldsymbol{A}^v$ and $\boldsymbol{B}^v$ are the left and right singular value matrices, respectively, obtained from the SVD of $\widetilde{\boldsymbol{L}}^v$.

The update rule for $\boldsymbol{R}^v$ is similar to that of $\boldsymbol{L}^v$, as described below:

$$\min_{\boldsymbol{R}^{vT}\boldsymbol{R}^v = \boldsymbol{I}} \left\|\boldsymbol{R}^v - \widetilde{\boldsymbol{R}}^v\right\|_F^2, \tag{9}$$

where $\widetilde{\boldsymbol{R}}^v = \left(\boldsymbol{Z}^v + \frac{\boldsymbol{Q}_2^v}{\mu}\right)^T\boldsymbol{L}^v\boldsymbol{C}^v$. The solution for $\boldsymbol{R}^v$ can be written as $\boldsymbol{R}^v = \boldsymbol{A}'^v\boldsymbol{B}'^{vT}$, where $\boldsymbol{A}'^v$ and $\boldsymbol{B}'^v$ are the left and right singular value matrices obtained from the SVD of $\widetilde{\boldsymbol{R}}^v$.

Step 4: $\boldsymbol{Z}^v$ can be obtained by optimizing the following problem with other variables held constant:

$$\begin{aligned} L(\boldsymbol{Z}^v) = &\left\|\boldsymbol{X}^v - \boldsymbol{X}^v\boldsymbol{Z}^v - \boldsymbol{E}^v + \frac{\boldsymbol{Q}_1^v}{\mu}\right\|_F^2 + \left\|\boldsymbol{Z}^v - \boldsymbol{L}^v\boldsymbol{C}^v\boldsymbol{R}^{vT} + \frac{\boldsymbol{Q}_2^v}{\mu}\right\|_F^2 \\ &+ \left\|\boldsymbol{Z}^v - \boldsymbol{S}^v + \frac{\boldsymbol{Q}_3^v}{\mu}\right\|_F^2, \end{aligned} \tag{10}$$

Taking the partial derivative of $\boldsymbol{Z}^v$ in Eq. (10) and setting it to zero, the update rule for $\boldsymbol{Z}^v$ is obtained as

$$\boldsymbol{Z}^v = \left(\boldsymbol{X}^{vT}\boldsymbol{X}^v + 2\boldsymbol{I}\right)^{-1}\left(\boldsymbol{X}^{vT}\boldsymbol{M}_1^v + \boldsymbol{M}_2^v + \boldsymbol{M}_3^v\right), \tag{11}$$

where $\boldsymbol{M}_1^v = \boldsymbol{X}^v - \boldsymbol{E}^v + \frac{\boldsymbol{Q}_1^v}{\mu}$, $\boldsymbol{M}_2^v = \boldsymbol{L}^v\boldsymbol{C}^v\boldsymbol{R}^{vT} - \frac{\boldsymbol{Q}_2^v}{\mu}$, and $\boldsymbol{M}_3^v = \boldsymbol{S}^v - \frac{\boldsymbol{Q}_3^v}{\mu}$.

Step 5: The subminimization problem for $\boldsymbol{S}^v$ can be rewritten as

$$\begin{aligned}
&\min_{\boldsymbol{S}^v} \sum_{i,j} p_{ij}^v \left\|\boldsymbol{x}_i^v - \boldsymbol{x}_j^v\right\|_2^2 s_{ij}^v + \frac{\mu}{2\lambda_1}\left\|\boldsymbol{Z}^v - \boldsymbol{S}^v + \frac{\boldsymbol{Q}_3^v}{\mu}\right\|_F^2 \\
&\quad = \sum_{i,j} p_{ij}^v d_{ij}^v s_{ij}^v + \frac{\mu}{2\lambda_1}\left\|\boldsymbol{S}^v - \left(\boldsymbol{Z}^v + \frac{\boldsymbol{Q}_3^v}{\mu}\right)\right\|_F^2 \\
&\quad = \mathrm{Tr}((\boldsymbol{P}^v \odot \boldsymbol{D}^v)^T\boldsymbol{S}^v) + \frac{\mu}{2\lambda_1}\left\|\boldsymbol{S}^v - \boldsymbol{M}^v\right\|_F^2 \\
&\text{s.t.} \ \ \mathrm{diag}(\boldsymbol{S}^v) = \boldsymbol{0}, s_{ij}^v \geq 0, \sum_j s_{ij}^v = 1,
\end{aligned} \tag{12}$$

where $\boldsymbol{M}^v = \boldsymbol{Z}^v + \frac{\boldsymbol{Q}_3^v}{\mu}$, $d_{ij}^v = \left\|\boldsymbol{x}_i^v - \boldsymbol{x}_j^v\right\|_2^2$, and $\odot$ represents the Hadamard product. Based on the result from [5], the above equation can be converted as

$$\min_{\boldsymbol{s}_i^v \geq 0, \boldsymbol{s}_i^{vT}\boldsymbol{1}=1, \boldsymbol{s}_{ii}^v=0} \left\|\boldsymbol{s}_i^v - \left(\boldsymbol{m}_i^v - \frac{\lambda_1 \boldsymbol{p}_i^v \odot \boldsymbol{d}_i^v}{\mu}\right)\right\|_2^2, \tag{13}$$

where $\boldsymbol{s}_i^v$ is the i-th column of matrix $\boldsymbol{S}^{vT}$ with similar definitions applying to $\boldsymbol{m}_i^v$, $\boldsymbol{p}_i^v$ and $\boldsymbol{d}_i^v$. To solve the optimization problem in (13), we can introduce the Lagrangian function:

$$L(\boldsymbol{s}_i^v, \alpha_i^v, \boldsymbol{\beta}_i^v) = \frac{1}{2}\left\|\boldsymbol{s}_i^v - \left(\boldsymbol{m}_i^v - \frac{\lambda_1 \boldsymbol{p}_i^v \odot \boldsymbol{d}_i^v}{\mu}\right)\right\|_2^2 + \alpha_i^v\left(1 - \boldsymbol{s}_i^{vT}\boldsymbol{1}\right) - \boldsymbol{\beta}_i^{vT}\boldsymbol{s}_i^v, \tag{14}$$

where $\alpha_i^v \geq 0$ and $\boldsymbol{\beta}_i^v \geq 0$ are the Lagrangian multipliers. Taking the derivative of Eq. (14) with respect to $\boldsymbol{s}_i^v$, we can obtain

$$\frac{\partial L\left(\boldsymbol{s}_i^v, \alpha_i^v, \boldsymbol{\beta}_i^v\right)}{\partial\left(\boldsymbol{s}_i^v\right)} = \boldsymbol{s}_i^v - \left(\boldsymbol{m}_i^v - \frac{\lambda_1 \boldsymbol{p}_i^v \odot \boldsymbol{d}_i^v}{\mu}\right) - \alpha_i^v\boldsymbol{1} - \boldsymbol{\beta}_i^v. \tag{15}$$

Applying the KKT condition in which $\boldsymbol{\beta}_i^v \cdot \boldsymbol{s}_i^v = 0$ (element-wise multiplication), the optimal solution can be derived as follows:

$$\boldsymbol{s}_i^v = \max\left(\boldsymbol{m}_i^v + \frac{\lambda_1 \boldsymbol{p}_i^v \odot \boldsymbol{d}_i^v}{\mu} + \alpha_i^v\boldsymbol{1}, 0\right). \tag{16}$$

Based on the constraint $\boldsymbol{s}_i^{vT}\boldsymbol{1} = 1$, we can obtain α_i^v as follows:

$$\alpha_i^v = \frac{1}{n} - \frac{1}{n}\sum_{j=1}^{n}\left(\boldsymbol{m}_{ij}^v - \frac{\lambda_1 \boldsymbol{p}_{ij}^v \odot \boldsymbol{d}_{ij}^v}{\mu}\right). \tag{17}$$

Step 6: When the other variables are fixed, $\boldsymbol{C}^v$ can be updated by

$$L\left(\boldsymbol{C}^v\right) = \frac{\mu}{2}\left\|\boldsymbol{Z}^v - \boldsymbol{L}^v\boldsymbol{C}^v\boldsymbol{R}^{vT} + \frac{\boldsymbol{Q}_2^v}{\mu}\right\|_F^2 + w^v\left\|\boldsymbol{C}^v - \boldsymbol{C}^*\right\|_F^2. \tag{18}$$

Algorithm 1 ADMM for solving scWDAC

Input: parameters λ_1, λ_2, and multi-omics feature matrices: $\{\boldsymbol{X}^1, \boldsymbol{X}^2, \cdots, \boldsymbol{X}^V\}$.
Output: Clustering results.
Initialization: Construct $\boldsymbol{P}^v$ uses KNN with Gaussian kernel function, $\boldsymbol{Z}^v = \boldsymbol{S}^v = \boldsymbol{C}^* = \boldsymbol{L}^v = \boldsymbol{C}^v = \boldsymbol{R}^v = \boldsymbol{Q}_2^v = \boldsymbol{Q}_3^v = \boldsymbol{0}_{n\times n}$, $\boldsymbol{E}^v = \boldsymbol{Q}_1^v = \boldsymbol{0}_{m_v\times n}$, $w^v = \frac{1}{V}$, $\varepsilon = 0.01$, $\mu = 0.01$, $\mu_{\max} = 10^8$, and $\rho = 2.8$.
while not converged and iter $<$ iter$_{\max}$
1. update $\boldsymbol{C}^*$ according to Eq. (4);
  for $v = 1 : V$
2. update $\boldsymbol{E}^v$ according to Eq. (6);
3. update $\boldsymbol{L}^v$ by solving problem (8);
4. update $\boldsymbol{R}^v$ by solving problem (9);
5. update $\boldsymbol{Z}^v$ according to Eq. (11);
6. update $\boldsymbol{S}^v$ by solving problem (13);
7. update $\boldsymbol{C}^v$ according to Eq. (19);
8. update w^v according to (21);
9. $\boldsymbol{Q}_1^v$, $\boldsymbol{Q}_2^v$, and $\boldsymbol{Q}_3^v$ are updated by Eq. (22);
  end
10. update μ by Eq. (22);
11. checking the convergence condition:
  $\max_{1\leq v\leq V} \left\| \boldsymbol{Z}^v - \boldsymbol{L}^v\boldsymbol{C}^v\boldsymbol{R}^{vT} \right\|_\infty < \varepsilon$.
end
12. compute $\boldsymbol{Z}^* = \sum_{v=1}^{V} \boldsymbol{Z}^v$;
13. apply the information reduction strategy to $\boldsymbol{Z}^*$;
14. compute the skinny SVD: $\boldsymbol{Z}^* = \boldsymbol{U}\varLambda\boldsymbol{V}^T$;
15. compute $\boldsymbol{M} = \boldsymbol{U}\sqrt{\varLambda}$;
16. construct the similarity matrix $\boldsymbol{W}$:
  $w_{ij} = \left(\frac{\boldsymbol{m}_i^T\boldsymbol{m}_j}{\|\boldsymbol{m}_i\|_2\|\boldsymbol{m}_j\|_2}\right)^2$;
17. perform spectral clustering on $\boldsymbol{W}$.

Computing the partial derivative of Eq. (18) concerning $\boldsymbol{C}^v$ and equating it to zero yields the update rule for $\boldsymbol{C}^v$:

$$\boldsymbol{C}^v = \left(\mu\boldsymbol{L}^{vT}\boldsymbol{N}^v\boldsymbol{R}^v + 2w^v\boldsymbol{C}^*\right)\left(\left(\mu + 2w^v\right)\boldsymbol{I}\right)^{-1}, \tag{19}$$

where $\boldsymbol{N}^v = \boldsymbol{Z}^v + \frac{\boldsymbol{Q}_2^v}{\mu}$.

Step 7: The optimization problem for w^v can be reformulated as

$$\min_{w^v} w^v \left\|\boldsymbol{C}^v - \boldsymbol{C}^*\right\|_F^2. \tag{20}$$

According to the result derived in [6], the above equation can be obtained as

$$w^v = \frac{1}{2\sqrt{\left\|\boldsymbol{C}^v - \boldsymbol{C}^*\right\|_F^2 + \delta}}, \tag{21}$$

where δ is set as 0.0001 to avoid dividing by zero.

Step 8: The Lagrange multipliers $\boldsymbol{Q}_1^v$, $\boldsymbol{Q}_2^v$, $\boldsymbol{Q}_3^v$, and the penalty parameter μ are updated as

$$\begin{cases} \boldsymbol{Q}_1^v = \boldsymbol{Q}_1^v + \mu(\boldsymbol{X}^v - \boldsymbol{X}^v\boldsymbol{Z}^v - \boldsymbol{E}^v), \\ \boldsymbol{Q}_2^v = \boldsymbol{Q}_2^v + \mu(\boldsymbol{Z}^v - \boldsymbol{L}^v\boldsymbol{C}^v\boldsymbol{R}^{vT}), \\ \boldsymbol{Q}_3^v = \boldsymbol{Q}_3^v + \mu(\boldsymbol{Z}^v - \boldsymbol{S}^v), \\ \mu = \min(\rho\mu, \mu_{\max}). \end{cases} \tag{22}$$

where μ and $\mu_{\max}$ are positive constants, and ρ determines the rate of convergence.

2 Evaluation Measurement

Two widely used metrics, Accuracy (ACC) and Normalized Mutual Information (NMI), are employed to evaluate the performance of scWDAC. ACC measures the consistency between the predicted labels and the actual labels by calculating the ratio of accurately predicted labels to the total number of cells. It is defined as

$$\mathrm{ACC}(\boldsymbol{T}, \boldsymbol{P}) = \frac{\sum_{i=1}^{n} \delta\left(t_i, \operatorname{map}\left(p_i\right)\right)}{n}, \tag{23}$$

where t_i and p_i represent the true labels and predicted labels for the i-th cell, respectively. n denotes the total count of cells. The function $\delta\left(x, y\right)$ equals 1 when $x = y$, otherwise it is 0. $\operatorname{map}\left(p_i\right)$ is a permutation mapping function that aims to map the predicted labels to the corresponding true labels using the Hungarian algorithm [7].

NMI is utilized to measure the similarity between two clusters. Assuming $\boldsymbol{Y}$ represents the predicted labels and $\boldsymbol{T}$ represents the true labels, NMI is defined as

$$\mathrm{NMI}(\boldsymbol{T}, \boldsymbol{Y}) = \frac{\sum_{i=1}^{n} \sum_{j=1}^{n} J_p\left(t_i, y_j\right) \ln\left(\frac{J_p(t_i, y_j)}{M_p(t_i) M_p(y_j)}\right)}{\sqrt{\left(\sum_{i=1}^{n} M_p\left(t_i\right) \ln M_p\left(t_i\right)\right)\left(\sum_{j=1}^{n} M_p\left(y_i\right) \ln M_p\left(y_j\right)\right)}}, \tag{24}$$

where $J_p\left(t_i, y_j\right)$ denotes the joint probability that an arbitrarily selected cell belongs to both cell types i and j, and $M_p\left(t_i\right)$ and $M_p\left(y_i\right)$ denote the probabilities of randomly selecting a cell belong to cell types i and j, respectively.

K-Nearest Neighbors Accuracy (KNA) is introduced as a metric to evaluate the effectiveness of cell classification in the embedding space using the K-Nearest Neighbors (KNN) algorithm, comparing it with the true class labels. KNA measures the consistency between the labels of a cell and its nearest neighbors in the embedding space. A higher KNA indicates better preservation of the local structure by the embedding algorithm. Assuming $\boldsymbol{T}$ represents the true labels of each algorithm and $\mathcal{N}_i$ denotes the set of nearest neighbors for cell i. KNA is defined as

$$\mathrm{KNA}(\boldsymbol{T}, \mathcal{N}) = \frac{1}{n} \sum_{i=1}^{n} \frac{1}{k} \sum_{j=1}^{k} \delta(T(\mathcal{N}_i(j)) = T(i)), \tag{25}$$

where k denotes the number of nearest neighbors, and the function $\delta\left(x, y\right)$ is an indicator that equals 1 when $x = y$ and 0 otherwise.

BS is proposed to quantitatively evaluate a method's ability to overcome the see-saw effect between ACC and NMI across multiple datasets. This metric explicity captures whether a method can consistently achieve high performance ini both metrics simultaneously, rather than trading one off for the other. For a method evaluated on D datasets, the BS is computed as:

$$\mathrm{BS} = \frac{1}{D} \sum_{d=1}^{D} \min\left(\mathrm{ACC}_d, \mathrm{NMI}_d\right) \tag{26}$$

where ACC_d and NMI_d denote the clustering accuracy and NMI on the d-th dataset, respectively.

3 The numerical comparison of ACC and NMI

The symbol "/" in S1 and S2 Tables indicates that the results are not obtained within the 60-hour execution limit or exceed the 256 GB memory capacity. Bold text in these tables highlights the best result for each dataset.

4 Clustering Visualization

The t-distributed stochastic neighbor embedding (t-SNE) algorithm [8] is a powerful technique for visualizing high-dimensional data in a low-dimensional space while preserving the relative distances between samples. To intuitively evaluate the clustering results, we use the learned similarity matrices of JSNMF, scAI, MVCLRS, and scWDAC on the SNARE, Inhouse, and PBMC datasets as input and apply t-SNE to project the predicted clustering results into two-dimensional space. The comparative visualization results are shown in S1 Fig.

For the SNARE dataset, which consists of four cell types: fibroblast cells (BJ), embryonic stem cells (H1), leukemia cells (K562), and lymphocyte cells (GM12878). S1 A Fig shows that JSNMF, MVCLRS, and scWDAC successfully separate the cells into four distinct clusters. However, there exists a certain degree of cell mixing, with scWDAC exhibiting the least cell confusion and MVCLRS producing more compact cell cluster distributions. In the visualization of the Inhouse dataset (S1 B Fig), the clusters identified by JSNMF and scWDAC exhibit well-separated and compact, whereas scAI and MVCLRS result in a mixture of different cell types, failing to achieve effective separation.

The PBMC (peripheral blood mononuclear cell) dataset contains seven distinct cell types: B cells, CD14+ monocytes, CD16+ monocytes, CD4+ T cells, CD8+ T cells, dendritic cells, and natural killer (NK) cells. No method can completely separate the clusters in the PBMC dataset visualization (S1 C Fig). However, compared to the other methods, scWDAC shows the least overlap between the identified clusters. JSNMF closely follows scWDAC in performance, while scAI and MVCLRS produce the poorest clustering results, with significant cell type overlap. These findings suggest that the similarity matrices learned by JSNMF and scWDAC are more effective in capturing the structural characteristics of data points in the low-dimensional subspace.

To further validate the performance of scWDAC, S2 Fig displays the block-diagonal structure of similarity matrices obtained by scWDAC and other methods. For the Sai dataset, the block structure of scWDAC is clearer than that of the other three methods. For the SNARE dataset, scWDAC and MALS exhibit clearer block-diagonal structures compared to RC_MSC and JSNMF. Since clearer diagonal blocks indicate better clustering and darker non-diagonal areas suggest a lower likelihood of cells from different types being assigned to the same subgroup. Therefore, scWDAC demonstrates superior performance in revealing more distinct clusters.

5 Analysis the effect of parameter settings

In this section, we primarily evaluate the impact of the regularization parameters λ_1 and λ_2, as well as the number of KNN neighbors k, on the performance of scWDAC. λ_1 and λ_2 represent the weights assigned to the weighted distance penalty term and the error term,

respectively. By treating ACC and NMI as functions of λ_1 and λ_2, and using a grid search technique within the range [5e-4, 1e-3, 3e-3, 5e-3, 8e-3, 1e-2, 3e-2, 5e-2, 7e-2, 1e-1, 5e-1], we evaluate the stability of scWDAC on four real multi-omics datasets.

As shown in S3 Fig, the ACC and NMI values for the Sai, Inhouse, and Mononuclear datasets show relatively stable variations, achieving superior clustering performance over a wide range of λ_1 and λ_2 combinations. This demonstrates the effectiveness and robustness of scWDAC. However, when the values of λ_1 and λ_2 are either too small or too large, clustering performance of scWDAC may deteriorate. For instance, a smaller λ_1 decreases the clustering performance on the Sai dataset, while a larger λ_1 also reduces the performance on the SNARE dataset. S4 Fig illustrates that when the KNN neighborhood size k is varied between 1 and 20, the ACC and NMI values for scWDAC remain stable across all eight datasets (with fluctuations $< \pm 0.025$). This stability demonstrates the robustness of the clustering results to changes in k. Therefore, to achieve optimal clustering result, we set $\lambda_1 = 0.01$, $\lambda_2 = 0.05$, and $k = 10$ for all datasets, as this configuration allows for superior clustering results on most datasets.

Additionally, to select the appropriate kernel function, we compare the linear, polynomial, Laplacian, and two Gaussian kernels with different bandwidths ($\sigma = 0.5$ and $\sigma = 1$) in S5 Fig. The results show that the Gaussian kernel ($\sigma = 1$) achieves the highest ACC and NMI on most of the test datasets, outperforming the other kernels and demonstrating the most robust performance.

6 Comparison of marker gene identification

As cellular characteristics and morphology are closely linked to gene expression, identifying marker genes is essential for understanding cell types and functions. In this section, we explore the performance of scWDAC in marker gene identification on the PBMC dataset using the COSG R package. S6 Fig presents the UMAP visualization of the PBMC dataset under four different labels. In S6 A Fig, cell clusters C1-C7 correspond to CD14+ monocytes, CD16+ monocytes, NK cells, CD4+ T cells, B cells, CD8+ T cells, and dendritic cells, respectively. These clusters in the UMAP plots correspond to the clusters shown in the marker gene identification subplots of S7 Fig.

S7 Fig and S6 Table present the results of marker gene identification based on true labels and predicted labels, from scWDAC, JSNMF, and scAI. As shown in S6 Table, scWDAC identifies 57 out of 70 true marker genes, significantly outperforming JSNMF (32 genes) and scAI (36 genes) in terms of overlap with the ground truth.

Moreover, the biological process enrichment analysis results, obtained using the cluster-Profiler tool and shown in S8 Fig, provide additional evidence supporting the effectiveness of scWDAC. Compared to the enrichment results from JSNMF and scAI, the marker genes identified by scWDAC exhibit a high degree of functional consistency with those derived from the true labels. This highlights the biological relevance and accuracy of scWDAC in marker gene detection.

7 Convergence analysis

Given the complexity of scWDAC, which involves multiple blocks, demonstrating its theoretical strong convergence is impractical. To empirically evaluate scWDAC's convergence, we

illustrate the objective value and clustering accuracy as a function of the number of iterations in S9 Fig. The objective value is calculated as

$$\text{Objective\_value} = \max_{1 \leq v \leq V} \left\| \boldsymbol{Z}^v - \boldsymbol{L}^v \boldsymbol{C}^v \boldsymbol{R}^{vT} \right\|_\infty .$$

S9 A Fig illustrates the relative errors over the number of iterations. It can be observed that scWDAC typically achieves convergence within 12 iterations, with the optimization process generally ranging from 1 to 30 iterations, demonstrating the effective convergence of our method. Additionally, the ACC and NMI values are calculated as a function of the number of iterations and are presented in S9 B Fig. The results show that both ACC and NMI values increase rapidly with the number of iterations and stabilize after about 12 iterations for most datasets, further demonstrating the effectiveness of the scWDAC optimization algorithm.

References

[1] Boyd S, Parikh N, Chu E, Peleato B, Eckstein J. Distributed optimization and statistical learning via the alternating direction method of multipliers. Found Trends Mach Learn. 2011;3(1):1–122. https://doi.org/10.1561/2200000016

[2] Mazumder R, Hastie T, Tibshirani R. Spectral regularization algorithms for learning large incomplete matrices. J Mach Learn Res. 2010;11:2287–2322. https://dl.acm.org/doi/10.5555/1756006.1859931 PMID: 21552465

[3] Yang J, Yin W, Zhang Y, Wang Y. A fast algorithm for edge-preserving variational multichannel image restoration. SIAM J Imaging Sci. 2009;2(2):569–592. https://doi.org/10.1137/080730421

[4] Schönemann PH. A generalized solution of the orthogonal procrustes problem. Psychometrika. 1966;31(1):1–10. https://doi.org/10.1007/bf02289451

[5] Wen J, Fang X, Xu Y, Tian C, Fei L. Low-rank representation with adaptive graph regularization. Neural Netw. 2018;108:83–96. https://doi.org/10.1016/j.neunet.2018.08.007 PMID: 30173056

[6] Nie F, Li J, Li X. Self-weighted multiview clustering with multiple graphs. InIJCAI. 2017;2564–2570. https://dl.acm.org/doi/abs/10.5555/3172077.3172245

[7] Kuhn HW. The Hungarian method for the assignment problem. Nav Res Logist Q. 1955;2(1-2):83–97. https://doi.org/10.1002/nav.20053

[8] Van der Maaten L, Hinton G. Visualizing data using t-SNE. Journal of machine learning research. 2008;9(11). http://jmlr.org/papers/v9/vandermaaten08a.html
